# Laying the groundwork for clinical teaching in Bachelor of Science Nursing programs: findings from a grounded Delphi study

**DOI:** 10.11604/pamj.2026.53.157.49660

**Published:** 2026-04-14

**Authors:** Grace Wangechi Gachuiri, Lucy Kivuti-Bitok, Miriam Wagoro

**Affiliations:** 1 Department of Nursing Science, University of Nairobi, Nairobi, Kenya

**Keywords:** BScN education, clinical placement, learning objectives, preparedness, simulation, skills lab, theory-practice integration

## Abstract

**Introduction:**

Bachelor of Science Nursing (BScN) students are exposed to clinical placements (CP) that are vital in advancing nursing competencies, but most of them attend the placements unprepared, thereby affecting learning, decision-making and safety. Even though CPs are compulsory, preparation is often unstructured and not consistent across universities hence undermining theory-practice integration. The research objective was to formulate systematic strategies that would enhance BScN students´ readiness for clinical placements.

**Methods:**

a 15-month Grounded Delphi study was conducted in three rounds: interviews with 13 professionals, followed by surveys with 103 and 75 participants (students, nurses, and lecturers). Thematic analysis and descriptive statistics were performed, and Kruskal-Wallis tests assessed differences in ratings of preparedness strategies across groups.

**Results:**

three key strategies emerged. The theory-to-practice Sequencing strategy emphasized acquiring theoretical knowledge before clinical exposure through coordinated instruction and essential skills training. The Goal-Oriented Learning Structures strategy focused on co-developed objectives and work plans using standard tools like rotation plans and logbooks. The Experiential Preparation approach highlighted simulations, mentorship, and practical training, though resource constraints remained a major challenge across universities and teaching hospitals.

**Conclusion:**

the academic-clinical interface in BScN programs can be strengthened with purposeful strategies aimed at preparing students with CPs by sequencing theory-practice, structured clinical learning with co-developed CP objectives, and experiential readiness through simulations. Nonetheless, the strategies must be implemented in a way that is viable to overcome the existing resource constraints.

## Introduction

The increasing concern over the clinical competence of newly graduated nurses [1-3], which is frequently associated with ineffective clinical placement (CP) [4-6], has cast doubts on the effectiveness of clinical rotations in equipping nursing students with the required practical competences [7-9]. Evidence shows that many Bachelors of Science Nursing (BScN) students attend their planned clinical placements unprepared, with poor procedural knowledge, vague role awareness, and poor confidence [1,10]. One study reported up to 40 percent of students were not prepared for clinical work exposure [11]. In another setting, students reported that their initial placements were emotionally overwhelming because of vague expectations and insufficient orientation [12].

CPs are supposed to harmonize theory and practice, but the actual practice experiences reveal the gaps in preparation strategies [13,14]. Some of the gaps include variability in curricula, inconsistent application of simulation, and lack of smooth integration between academic teaching institutions and clinical teaching institutions [15,16]. Consequently, poorly prepared students risk jeopardizing not only patient and health worker safety [14,17-19] but also burden clinical teams [20,21], and miss critical learning opportunities [22-25]. Therefore, there is a need for organized, standardized and supportive measures that prepare and equip students to enter the real-life healthcare environment [26-28].

To fill the gaps, the literature offers three important processes that facilitate readiness [29-31], beginning with the cognitive priming strategy where theoretical training precedes practice. The second strategy is the development and dissemination of rotation-specific learning objectives [32] to promote purpose-directed learning. Last but not least, the skills lab-based training in the form of simulation-based practice and case presentations that provide secure settings of experiential preparedness [33].

Regulators like the Nursing Council of Kenya insist on CPs guided by exposure hours, but they do not directly mandate practices that promote student readiness for CPs since universities have discretion in designing and implementing curricula [23]. Consequently, practices that support student readiness for CPs are not consistently applied in clinical settings [34,35]. Such discrepancy, evident not only in Kenya but also in other countries, may be aggravated by the limited coordination between academic institutions and clinical facilities, which operate independently of each other [21,36-38]. The central issue is not whether clinical placements are either necessary or available, but whether preparations are made to ensure that students are cognitively and procedurally prepared and whether there are structures that are deliberately put in place to facilitate the process.

Preparedness should not be a mere pedagogical addition to clinical placements but a systemic requirement [36,39] that should be established and not presumed. Clinical teaching continues to depend on unstructured exposure [40], and students are supposed to adapt to clinical exposure and not necessarily be intentionally primed for maximum learning and participation [32,34,36,41]. In other cases, students are sent for CPs with little to no instructions [39], vague expectations [12], and poor exposure to procedures [33], a problem that is exacerbated by overcrowded wards and undertrained preceptors [42]. Thus, the research examined preparedness as a foundational aspect of BScN clinical teaching to develop structured approaches that can be used to promote preparedness prior to placements. The article presents the research methods, the findings, the discussion, the conclusion and implications of the study.

## Methods

The Grounded Delphi methodology was applied in a 15-month study, which combined both qualitative and quantitative approaches to establish and formulate consensus on the CPs preparedness strategies.

### Study setting and participants

The study targeted universities and teaching hospitals that are directly engaged in clinical education of undergraduate nursing students between May 2023 and August 2024. These were 22 accredited universities with BScN programs and their affiliated clinical placement teaching hospitals. There has been an increased interest in the quality of nursing education in Kenya, where governance of the healthcare industry is decentralized amid a rising number of diverse training institutions.

The participants comprised people who had substantive roles in undergraduate nursing education and clinical supervision. These were university lecturers (BScN program), clinical nurses who supervised students on placements and final-year BScN students who had a minimum of three years of experience in placements. The selection aimed to attract those with informed views and preparedness for CPs from both academic and practice settings.

Snowballing was used in round 1, where 13 experts were interviewed. The qualification criteria were post-basic education in nursing and at least five years of supervision experience. These experts were recruited through the faculty offices and nurse managers in the 22 universities and teaching hospitals. The first interaction was through phone calls and follow-up emails with study documents, including consent forms.

Round 2 involved a wider group, which was purposely recruited using faculty office heads and nurse managers. The recruitment was directed at stakeholders who actively participated in clinical placements for BScN programs. A total of 103 respondents (lecturers, clinical nurses, and students) filled the online questionnaire. In round 3, only 75 responses were received (73% response rate), although stakeholder diversity was retained. The third round was used to provide validation of the previous findings (Table 1).

**Table 1:** profile of Delphi participants across rounds 2 and 3

Participant characteristic		Round 1 (N=13)	Round 2 (N=103)	Round 3 (N=75)
1. Occupation	4th year BSc Nursing student	-	27 (26%)	15 (20%)
	Clinical Area Nurse	3 (23%)	18 (17%)	19 (25%)
	Other occupation	-	2 (2%)	0 (0%)
	University Lecturer	10 (77%)	57 (55%)	41 (55%)
2. Age	<25 years	-	22 (21%)	8 (11%)
	25-34 years	-	15 (15%)	18 (24%)
	35-44 years	9(69%)	47 (46%)	34 (45%)
	45-54 years	3(16))	16 (16%)	14 (19%)
	>55 years	2(15%)	3 (3%)	1 (1%)
3. Gender	Female	10(77%)	62 (60%)	44 (59%)
	Male	3(23%)	41 (40%)	31 (41%)
4. Years of experience	Below 5 years	-	18 (17%)	12 (16%)
	5 -15 years	6 (46%)	50 (49%)	40 (53%)
	16 - 24 years	4(31%)	7 7%)	9 (12%)
	25 - 35 years	3(23%)	5 (5%)	3 (4%)
	None (pre-service students)	-	27 (26%)	1 (1%)

Distribution of participants by occupation, age, gender, and years of experience in round 2 (N = 103) and round 3 (N = 75) of the Delphi process.

### Methodology, method, and study design

A constructivist qualitative methodology was applied to explore preparedness for clinical placements in undergraduate nursing education, which is an appropriate approach in the creation of context-specific knowledge in dynamic learning environments [37,38]. The hybrid (Grounded Delphi) design involved the combination of grounded theory and Delphi methods [39]. Round 1 involved an open-ended exploration using grounded theory, while round 2 and round 3 were surveys that used structured and iterative rounds guided by Delphi principles. This mixture allowed both in-depth exploration and consensus building, which is in line with the exploratory and practice-based objectives of the study.

Round 1 was a semi-structured interview with professionals, which was transcribed and thematically coded in order to determine student preparedness strategies. The second and third rounds used online Likert-scale questionnaires to evaluate the significance and feasibility of these strategies. Descriptive analysis of quantitative data was done, and the comments emanating from open-ended questions provided complementary insights.

The grounded Delphi design was used to convert the expert views to practical recommendations. Round 1 created high-level strategies, round 2 helped to generalize them into survey items rated in terms of feasibility and significance, while round 3 gave controlled feedback allowing participants to reconsider and reach consensus. The anonymity of participants was ensured to the end in order to decrease bias and promote independent judgment. The Delphi design principles of diversity and consensus saturation were used to determine sample size.

### Data collection procedure

In round 1, in-depth interviews were held with 13 experts through face-to-face or online platforms depending on the availability of participants. The interview guide explored perceptions, challenges, and strategies related to student preparedness for CP. Interviews were recorded (with consent), transcribed word-for-word, and analysed concurrently with data collection until thematic saturation was achieved.

In round 2, the results of round 1 were summarized into 38 strategy statements, sorted by themes and measured on a 5-point Likert scale (1-strongly disagree to 5-strongly agree). Besides, opportunities for open-ended feedback were provided. The preparedness strategies were operationalized as those actions or mechanisms that were believed to enhance the students in their capacity to be effective in their clinical learning. The online survey was distributed to 126 individuals, whereby 103 (82%) responded to it. Responses were shared through a private and secure system.

The results of round 2 were summarized with median scores. Strategies that did not reach a consensus (=75%) were retained for a re-evaluation in the next round. During round 3, participants were given feedback that included previous ratings and group statistics and re-rated each item. Out of 103 invited, 75 (73%) individuals completed the final round.

### Data analysis

Analysis followed a sequential Grounded Delphi approach, with qualitative findings guiding quantitative instrument design. Qualitative data were analyzed using constructivist grounded theory. Transcripts underwent initial, focused, axial, and theoretical coding to build a thematic framework. Coding was supported by Delve software, with two researchers coding independently and meeting regularly for consistency. Preparedness strategies were operationalized as actions or mechanisms perceived to improve students' ability to engage effectively in clinical learning, measured using a 5-point Likert scale (1-strongly disagree to 5-strongly agree). Final themes informed the round 2 structured survey.

Quantitative data from rounds 2 and 3 were analyzed in SPSS. Descriptive statistics (median, interquartile range, and percentage agreement) assessed consensus, predefined as =75% rating an item 4 or 5. Non-consensus items from round 2 were re-rated in round 3 after group feedback. All survey questions were mandatory, no missing data occurred. Participant attrition from 103 (round 2) to 75 (round 3) was attributed to competing professional obligations and reflected normal Delphi participation decline. Kruskal-Wallis tested differences across students, clinical nurses, and lecturers; p>0.05 indicated consistent ratings across groups. No sensitivity analysis was performed, as consensus levels were the primary endpoint.

### Trustworthiness

Trustworthiness followed Lincoln and Guba´s (1985) criteria: credibility, dependability, confirmability, and transferability. The Delphi iterative process was used to build credibility through ratings and feedback to polish the strategies into a context-relevant model. Reliability was facilitated by a uniform analytic scheme and thorough documentation. Confirmability was based on audit trails and memos of the analytic decisions. The transferability was also improved by providing descriptive information about the participants and study setting, as it could help evaluate whether the model could be applied to other learning environments. Other than this, power influence was minimized by keeping anonymity in the rounds.

### Ethical considerations

Ethical review and approval were obtained from the Kenyatta National Hospital-University of Nairobi Ethics and Research Committee through approval number KNH-ERC/A/233. A permit was also obtained from the National Commission of Science, Technology and Innovation (NACOSTI License No.: NACOSTI/P/23/28224). The involved institutions provided administrative permission. All participants were informed and signed the informed consent. Data were coded and stored in a safe place.

## Results

The study aimed to develop and validate strategies for enhancing preparedness for CPs among BScN students in a three-round Delphi process. The results are presented in a chronological order of data collection rounds, starting with findings from in-depth interviews that produced qualitative themes, then the stakeholder ratings and consensus outcomes of round 2 and round 3. The results reflect the breadth of expert views as well as the depth of congruence among participating stakeholder groups.

### Identification of preliminary strategies (round 1)

Five themes emerged to describe strategies that could enhance preparedness as a means of enhancing the experience and learning of BSc Nursing students during clinical placement. First is the need to align theory and practice through integrated curricula and harmonised instruction methods. Second, the need for structured orientation with early communication of objectives to all clinical teaching stakeholders. Third, structuring clinical learning via co-developed, placement-specific objectives. Fourth, enhanced experiential readiness through skills laboratory practice, simulations, and return demonstrations to build confidence in students prior to patient encounter. Fifth, use of critical tools such as rotation plans, logbooks, and manuals that standardize supervision and ensure continuity between academic and clinical settings. Table 2 provides participant descriptions illustrating the themes and subthemes.

**Table 2:** themes, subthemes, and illustrative quotes from round 1

Theme	Subtheme
1. Aligning theory and practice	Harmonized teaching methods
	Integration of theory and practice (block or integrated)
	Collaborative learning among students
2. Orientation	Early orientation to objectives
	Physical orientation to clinical sites
	Faculty presence on first day
	Nurse clinician orientation/capacity building
3. Structuring clinical learning	Participatory development of objectives
	Objectives specific to placement area
	Realistic, non-repetitive objectives
	Collaborative Work planning and joint schedules
4. Simulation and experiential readiness	Skills lab practice
	Case studies and return demonstrations
	Infrastructure to support learning
5. Critical tools	Master rotation plans
	Logbooks, checklists, procedure manuals

### Refinement and prioritization of strategies (round 2)

Round 2 clarified round 1 strategy by translating it into structured survey items for stakeholder review. Responses showed strong agreement, with mean scores between 4.1 and 4.8. High ratings emphasized the importance of clear communication and preparation, particularly orientation to the clinical area (mean = 4.8), discussion of objectives between the students, clinical nurses, and the lecturers (mean = 4.75), and supervisors identifying learning opportunities that relate to objectives to be met (mean = 4.63). Experiential methods also received strong endorsement, including peer learning (mean = 4.55) and task-based learning (mean = 4.54).

When asked to suggest additional preparation before placements, respondents highlighted psychological and practical readiness through theoretical exposure, skills-laboratory practice, and clear objectives. However, these views largely echoed round 1 suggestion, rather than introducing new strategies. For example:

“*The course content that has already been covered. Learners should be placed in a clinical area where they have theoretical experience. The support tools, like charts, stationery, and applicable clinical tools, should be available. Preceptors should be prepared in advance with their work spelt out*” (P39).

“*Clearly stating the objectives to be met by the learner and how the lecturer is going to assist in meeting these expectations*” (P42).

“*The student nurses ought to be taught the various nursing skills in the skills lab prior to the placement so that they can get to actualize what they learnt in the lab*” (P14).

The insights from round 2 led to a refined framework that outlined the major CP preparedness strategies. Based on those findings, round 3 was aimed at validating the framework through appraisal of stakeholder roles and responsibilities in the implementation of the proposed strategies. This last step enhanced the empirical validity and contextual relevance of the framework to the nursing education practice.

### Final validation of strategies (round 3)

Round 3 validated the refined CP preparedness strategies by examining the stakeholder views on the application of the proposed measures. Faculty, clinical nurses and students were able to define their respective roles in preparedness for CP. The three validated strategies are presented.

### Theme 1: sequencing of theory and clinical practice

The results of round 3 indicated that all stakeholders highly supported a systematic, gradual process of transitioning classroom learning to clinical practice facilitated by system-level coordination. The participants noted that there should be a collective responsibility of the faculty, students, and nurses in ensuring that theory and practice are aligned. It was considered necessary to ensure that the prerequisite knowledge and professional behaviours were met prior to clinical placement (mean = 3.61). High level of agreement was achieved on the issue of strengthening the university-hospital collaboration by means of communication (4.1) and mentoring programs (mean = 3.73), better placement scheduling to minimize congestion (mean = 4.25) and professional development to improve supervision and appraisal capacity of clinical nurses (mean = 3.56).

It was also recommended that pre-placement preparation should involve peer learning by the use of role-plays and returned demonstrations (mean = 3.73). On-site implementation was focused on coordinated involvement during the initial day of placement. Students should be accompanied by a lecturer on their first day in a new placement (mean = 3.40), oriented (mean = 3.85) and assigned a nurse mentor (mean = 3.41). Faculty involvement in the ward orientation (mean = 3.59) and the presentation of the learning objectives to the ward staff by faculty (mean = 3.63) were also regarded as important issues to facilitate the transition and alignment of academic and clinical learning.

### Theme 2: structuring clinical learning through objectives and work planning

This theme was organized according to the Triple S Approach (Setting, Synthesising, and Sharing Objectives) to provide systematisation that enables the alignment of learning goals with clinical opportunities. Faculty set behavioural and professional expectations (mean = 3.56), students are actively involved in setting objectives of the placements (mean = 3.33) and personal learning objectives (mean = 3.53). The engagement of faculty, students, and clinical nurses would encourage a sense of collective ownership and responsibility, whereby the goals are addressed to the needs of the institutions and each student individually.

In synthesising objectives, students are expected to interpret objectives into competencies (mean = 3.56), develop work plans as learning roadmaps (mean = 3.67), and align them with personal goals (mean = 3.60). Clinical nurses review objectives (mean = 3.44), identify relevant learning opportunities (mean = 3.47), and guide students toward suitable activities (mean = 3.16). Faculty complement this by proposing structured activities (mean = 3.59), following instructional plans (mean = 3.45), and coordinating nurses´ supervision (mean = 3.25).

Sharing Objectives and Work Plans underscores collaboration to ensure a common understanding among all participants. Objectives are jointly discussed by students with lecturers (mean = 3.68), clinical nurses with lecturers (mean = 3.55), and students with nurses (mean = 3.55). Faculty reinforce this process by aligning clinical opportunities with learning goals (mean = 3.55) and facilitating communication across groups (mean = 3.63). Such collaboration enhances coherence in supervision and promotes consistent learning experiences throughout the clinical placement process.

### Theme 3: building competence through mentorship and practice

This theme highlights the importance of simulation and experiential learning in preparing students for clinical placements. Stakeholders emphasized simulation in skills labs for observation, guided practice, and refinement (mean = 3.73). Mentorship, through faculty demonstrations (mean = 3.53) and continuous clinical supervision (mean = 3.41), was viewed as crucial for bridging theory and practice. They also recommended professional development for clinical nurses (mean = 3.56) and adequate resources to support simulation and clinical learning.

“*Then another thing is that when you have a big population of students there is also the challenge of resources... For example, if I can talk about a simple thing like gloves, you find the stocks are depleted very fast because the number of students is very high. And therefore because of the challenges of resources, some students may miss to practice certain procedures..*.” (P4).

“*Preparing students for clinical placement is a bit tricky because, for instance, the issue about the procedures and the guidelines. The procedures touch on the resources. If you don´t have gloves, linen… (yeah), there are some things unless you have the resources you cannot do (practice) them the right way*” (P2).

“*Make sure that the things that the students would utilize in (clinical) learning in terms of procedures and learning skills are in place. Because there are many times, we send students to the wards and the units, the patients are there yes, but sometimes they may lack the necessary things that should be there for them to learn the skills. So, one ought to prepare the ground for them*” (P4).

### Stakeholder consensus and differences

Consensus was strong across stakeholder groups on sequencing theory with clinical practice, showing broad support for structured communication and system-level preparation of students. Learning objectives, work plans, checklists, and clear faculty-nurse roles were consistently rated essential for guiding and assessing learning (p = 0.068-0.868). Agreement also emerged on simulation and experiential readiness, with mentoring programs (p = 0.351) and faculty demonstrations (p = 0.500) valued for improving preparedness. However, divergence arose regarding aligning work plans with individual student objectives (p = 0.002), as students and nurses favored flexibility while lecturers preferred standardization (Table 3).

**Table 3:** stakeholder ratings of preparedness strategies across professional groups

Question		Total (N=103)	4^th^ year BSc Nursing student (N=27)	Clinical Area Nurse (N=17)	Other Degree (N=2)	University Nursing Lecturer (N=57)
Rate the degree to which the following activities could be helpful in preparing learners to own and drive the learning process while in the clinical area for placement	Engage learners while developing clinical learning objectives	4.25	4.52	4.18	4	4.16
	Share already developed objectives with learners	4.32	4.44	3.82	5	4.39
	Learners discuss clinical objectives with lecturer	4.75	4.74	4.71	5	4.75
	Involve learners in breaking down objectives into desired clinical competencies	4.56	4.63	4.18	4.5	4.65
	Allow learners to make choice of the hospital of placement among the available options	3.14	3.33	3.35	4.5	2.93
	Get orientation of the clinical area environment	4.8	4.78	4.71	5	4.82
	Learners draw a work plan to guide their learning activities as per the clinical placement objectives	4.62	4.67	4.82	4.5	4.54
	Engage learners to prepare a checklist for gauging performance in required competencies	4.12	4.26	4.06	2.5	4.12
Rate the extent to which you agree that the following initiatives can help to optimise learning in a student-centred approach of clinical teaching	Allowing learners to initiate the subject to be learnt	3.25	3.41	2.76	4.5	3.28
	Learner develop individual learning goals	3.98	3.89	3.71	5	4.07
	Having peer learning activities	4.55	4.41	4.35	5	4.67
	Supervisors do what learners can do during supervision rounds.	3.67	4.04	3.06	2	3.74
	Tasking learners with a responsibility to develop/create something	4.54	4.41	4.59	4.5	4.6
	Learners adhering to their work plan	4.59	4.59	4.41	5	4.63
	Learner Identify learning opportunities available at the clinical site	4.63	4.48	4.59	5	4.7
	Keep a journal of daily clinical experiences	4.55	4.3	4.47	5	4.68

Mean ratings (on a 5-point Likert scale) of proposed preparedness strategies by stakeholder category during round 2 of the Delphi study (N = 103). Higher scores indicate stronger agreement or perceived helpfulness in preparing students for clinical placements.

## Discussion

This study used a grounded Delphi approach to examine BScN student preparedness for clinical placements. It identified three key strategies: sequencing theory with practice, structuring learning through objectives and work plans, and enhancing experiential readiness through simulation. Preparedness emerged as a co-produced process requiring collaboration among students, faculty, and clinical nurses. The participatory design strengthened credibility and consensus, providing a strong foundation for developing indicators of preparedness in BScN education.

The results support the existing evidence that underscores the significance of matching classroom and clinical training of reducing anxiety and improving cognitive preparedness [6,25]. They also support the ‘clinical cognitive alignment’ [40] model, which underlines the importance of starting with theory, then practice. In concurrence with the other research conducted in Ghana [20], the study finds that accountability and improved performance of students can be achieved through structured learning goals and common work plans. In contrast to reports of educator resistance to objective-based approaches due to weak institutional communication [41], this study found convergence between academic and clinical perspectives, suggesting that dialogue and reflection can overcome resistance.

Differing opinions were observed among the stakeholder groups. Flexibility and focus on individual goals were more important to the students and nurses, and the faculty focused on standardisation, potentially because they were more accustomed to curriculum design [42]. This mismatch emphasizes the importance of negotiation and joint planning. Also, the differences in the perceptions of experiential readiness across institutions might be attributed to the variable familiarity with simulation pedagogy [32] and resource limitations.

The combined framework makes preparedness a system-level construct that connects sequencing, structured learning and simulation along a continuum. It highlights the fact that preparedness is achieved through coordinated university-hospital collaborations as opposed to unilateral interventions. This view builds on previous research that viewed simulation as a skills acquisition tool [43], but places it in a context of a wider pedagogical system that aids in the development of professional identity. These strategies ought to be implemented through alignment of the curriculum, development of the faculty, and inter-organisational cooperation to provide uniform, competency-based education [44].

The findings should be interpreted cautiously due to self-reported data, contextual differences, and a focus on consensus rather than measurable outcomes. They may apply to BScN programs in sub-Saharan Africa with similar curricula and resources. Future research should examine how preparedness strategies influence competence, confidence, and patient safety through longitudinal and comparative studies to identify contextual factors affecting students´ transition to independent practice.

### Limitations

The research relied on self-reported perceptions as opposed to direct observation, and this could have created bias in responses. At the data collection period, only 22 universities were active in Kenya, and this restricts the representativeness of all BScN programs currently. The Delphi design encouraged consensus, but it might have been biased towards divergent opinions. These limit the generalizability, but the study provides useful, situation-specific information and a useful framework on how to enhance clinical preparedness plans within BScN programs.

## Conclusion

BScN programs embrace effective clinical placements through structured preparation to foster intentional learning, safe practice, and professional competence. The tested framework highlights three interrelated priorities, which include sequencing theory and practice, aligning learning with work plans, and enhancing simulation and experiential preparedness. Clear coordination among students, faculty, and clinical nurses bridges gaps that disrupt learning and patient safety. Structured, mentored approaches can transform unplanned exposure into consistent, competency-based experiences that build confidence and professional identity. The framework is suitable for curriculum development and academic-clinical partnerships, positioning intentional preparedness as an educational innovation and a strategic investment in producing capable, safe, and competent practitioners who contribute meaningfully to healthcare quality and system capacity.

### What is known about this topic

- Even though the majority of BScN programs describe the clinical learning objectives, they are not implemented uniformly because of the lack of adequate collaboration and coordination among educators, preceptors and students;
- The absence of standard models of preparation for clinical placements contributes to the inconsistency in the translation of clinical objectives into learning, teaching and supervision practice;
- The differences in preparation for clinical placements across institutions lead to disproportionate student learning opportunities and competence attainment by the end of a clinical placement.

### What this study adds

- This study develops a context-specific model that lays the groundwork for standardized, evidence-informed clinical teaching in resource-limited settings;
- It employs a participatory, stakeholder-driven approach integrating perspectives of students, clinical nurses, and faculty to enhance practicality and ownership;
- It provides a structured checklist to guide consistent planning, delivery, and evaluation of clinical learning across nursing programs.
